# LONG-TERM PATTERNS OF FAMILY AND PAID CAREGIVING BY CARE TYPES AND SOURCES

**DOI:** 10.1093/geroni/igae098.3866

**Published:** 2024-12-31

**Authors:** Esther Shin, Sojung Park, Soobin Park, BoRin Kim, Eunsun Kwon, Seoyeon Ahn

**Affiliations:** Illinois State University, Normal, Illinois, United States; Washington University in St. Louis, St. Louis, Missouri, United States; Washington University in St. Louis, St. Louis, Missouri, United States; University of New Hampshire, Durham, New Hampshire, United States; Fairleigh Dickinson University, Hackensack, New Jersey, United States; Duke-NUS Medical School, Singapore, Singapore

## Abstract

To support aging in place for community-dwelling older adults, sustainable long-term care and support are crucial. The existing literature often focuses on family caregiving, formal caregiving, or their relationship, such as whether they substitute for each other. This study aims to broaden the understanding of collaborative care by exploring the intersection of different care forms and caregiver sources, an area that is less studied. Using data from the National Health and Aging Trends Study (2011-2022), this research examines longitudinal patterns of caregiving, focusing on the types of services (ADL/IADL support) and their sources (unpaid informal, paid formal, and paid family caregivers). The study sample included individuals who received consistent caregiver support throughout the study period. Group-based multiple trajectory (GBMT) modeling identified four distinct care mix patterns: (1) consistently low care (72.2%), (2) increasing ADL informal care (18.3%), (3) increasing formal care (7.4%), and (4) decreasing formal care with increasing paid informal care (2.1%). Compared to the relatively healthy and less vulnerable “consistently low care” group, those receiving “increasing ADL informal care” were more likely to be married women, while those with “increasing formal care” tended to live alone, be male, and have dementia. Notably, individuals with “decreasing formal care but increasing paid family care” were often racial minorities, in poor health, and older, possibly utilizing consumer-directed care programs. These findings provide an initial empirical foundation for understanding care mix, highlighting the need for further research on gender-specific caregiving patterns and the impacts of these care trajectories on health outcomes.

